# GWAS-identified *CCR1* and *IL10* loci contribute to M1 macrophage-predominant inflammation in Behçet’s disease

**DOI:** 10.1186/s13075-018-1613-0

**Published:** 2018-06-12

**Authors:** Hiroto Nakano, Yohei Kirino, Mitsuhiro Takeno, Kana Higashitani, Hideto Nagai, Ryusuke Yoshimi, Yukie Yamaguchi, Ikuma Kato, Ichiro Aoki, Hideaki Nakajima

**Affiliations:** 1 Yokohama City University Graduate School of Medicine, Department of Stem Cell and Immune Regulation, Yokohama, Japan; 2 Nippon Medical School Graduate School of Medicine, Department of Allergy and Rheumatology, Tokyo, Japan; 3 Yokohama City University Graduate School of Medicine, Department of Environmental Immuno-Dermatology, Yokohama, Japan; 4 Yokohama City University Graduate School of Medicine, Department of Molecular Pathology, Yokohama, Japan

**Keywords:** Behçet’s disease, Macrophage polarization, GWAS, CCR1, IL-10

## Abstract

**Background:**

Low C-C chemokine receptor 1 (CCR1) and interleukin (IL)-10 expression is associated with risk of Behçet’s disease (BD). The objective of the present study was to clarify the pathological roles of *CCR1* and *IL10* loci identified by previous BD genome-wide association studies (GWASs).

**Methods:**

M1 and M2 macrophages (Mφ) were differentiated with granulocyte-macrophage colony-stimulating factor or macrophage colony-stimulating factor (M-CSF) from peripheral monocytes of healthy control subjects (HC) and patients with BD. Expression of CD68 and CD163 was evaluated to test for Mφ polarization. CCR1 and IL-10 messenger RNA (mRNA) and protein expression was compared according to *CCR1* and *IL10* single-nucleotide polymorphism (SNP) genotypes. The migratory ability of M1 and M2 Mφ toward CCR1 ligand macrophage inflammatory protein (MIP)-1α was compared. The ratio of M1 and M2 Mφ in skin lesions of BD and systemic sclerosis (SSc), which was reported to be M2 Mφ-dominant, was compared. To examine the plasticity of polarized Mφ, the differentiated cells were cultured with either the same or the other culture condition.

**Results:**

Preferential expression of CD163, CCR1, and IL-10 was found in M2 Mφ compared with M1 Mφ. M2 Mφ migrated more sensitively to low concentrations of MIP-1α than M1 Mφ did. BD-derived M1 Mφ showed higher CCR1 surface expression than HC-derived M1 Mφ did. *IL10* and *CCR1* mRNA expression differences were observed by GWAS-identified SNP genotypes in polarized Mφ. BD skin lesions showed M1 Mφ predominance compared with SSc skin lesions. A plasticity assay revealed that M-CSF restored IL-10 synthesis and reduced IL-6 production by M1 Mφ.

**Conclusions:**

The present study reveals that GWAS-identified SNPs contribute to M1 Mφ-predominant inflammation in BD. Our data also suggest that the skewed Mφ polarization is correctable by immunological intervention.

**Electronic supplementary material:**

The online version of this article (10.1186/s13075-018-1613-0) contains supplementary material, which is available to authorized users.

## Background

Behçet’s disease (BD) is an idiopathic inflammatory disease that affects multiple organs, including the skin, eyes, vasculum, and mucosa [1, 2]. Genetic factors such as HLA-B*51, as well as environmental factors, including microbial components, have been implicated in the pathogenesis of BD [3]. Recent genome-wide association studies (GWASs) followed by subsequent genetic studies have identified nearly 20 loci as the disease susceptibility genes, most of which are involved in both innate and acquired immune systems [4–10]. Of them, *IL10* and *CCR1* are highly expressed genes in macrophages (Mφ). IL-10 is one of the cytokines conferring the anti-inflammatory effect, involved in the pathogenesis of various autoimmune diseases, including systemic lupus erythematosus (SLE) and inflammatory bowel diseases (IBD) [11]. Some studies have shown evidence that IL-10 is increased in BD inflamed tissues [12, 13]. However, genetic studies revealed that a BD risk single-nucleotide polymorphism (SNP) allele is associated with lower interleukin (IL)-10 production [4, 6], implicating complex interaction of T-helper cell Th1, Th2, and Th17 cytokines in BD [14].

CCR1 (C-C chemokine receptor 1), a membrane receptor for chemokines such as macrophage inflammatory protein (MIP)-1α, is known to be expressed on myeloid cells and is involved in cell migration [15]. We previously reported that the disease risk allele of *IL10* locus rs1518111 is associated with reduced production of IL-10, as well as of *CCR1* locus rs7616215, an SNP in the 3′ noncoding region, and that it is associated with lower *CCR1* messenger RNA (mRNA) expression and reduced monocyte chemotaxis [5]. The result was unexpected because the low chemotactic ability of monocytes, which seems to be protective from inflammation, was identified as a risk for BD. This genetic findings do not directly correspond to previous functional studies that have shown excessive production of Mφ-derived proinflammatory cytokines, including tumor necrosis factor (TNF)-α, which is the most effective therapeutic target in BD [16, 17]. Additionally, our recent genetic study suggested that an effective target may be the IL1A–IL1B locus, which is related to enhanced IL-1β production from activated Mφ [10].

Mφ polarization concepts have gained attention, especially in the field of tumor immunity [18]. Further studies have revealed that Mφ are divided into two major subtypes with distinct functions: M1 and M2 Mφ. M1 Mφ are classical Mφ and play a proinflammatory role through the production of IL-6 and TNF-α, and they activate the Th1 cellular immune response, whereas M2 Mφ are nonclassical Mφ which produce anti-inflammatory cytokine IL-10 that suppresses inflammatory responses [19]. The functional and phenotypic diversity of Mφ has been also investigated in physiological and other pathological conditions [20]. Recent GWASs have identified *IL10* and related genes as susceptibility genes associated with immune-mediated diseases such as IBD and SLE in addition to BD, suggesting the involvement of abnormal M2 Mφ function in the pathogenesis of these diseases [21]. Indeed, dysfunction of M2 Mφ has been shown to exacerbate inflammation in a herpes simplex virus-induced BD mouse model [22]. We previously revealed that downregulation of heme oxygenase (HO)-1, which is preferentially expressed as an M2 Mφ-specific anti-inflammatory protein, suggested M2 Mφ dysfunction in BD [23]. These findings prompted us to analyze functional studies of Mφ polarization in human patients with BD. The results of the present study support the hypothesis that GWAS-identified *CCR1* and *IL10* loci contribute to impaired M2 Mφ function, which is partly due to genetic predisposition, and lead to M1 Mφ-predominant immune responses in BD.

## Methods

### Study subjects

All of the individuals with BD in this study fulfilled the International Study Group (ISG) criteria for BD [24]. The study protocol and genetic analyses were approved by the Yokohama City University Hospital Ethics Board (A140327015 and B131107009). All of the human samples were used after written informed consent was obtained.

### Cell preparation and culture

Peripheral blood mononuclear cells (PBMCs) were isolated by density gradient centrifugation using Lymphoprep (Axis-Shield, Dundee, UK). Human monocytes were isolated from PBMCs using the Human Monocyte Isolation Kit II magnetic-activated cell sorter (Miltenyi Biotec, Bergisch-Gladbach, Germany) according to the manufacturer’s protocol [23]. Isolated monocytes were plated at a density of 5 × 10^5^ cells/ml and were incubated in RPMI 1640 medium (R8758; Sigma-Aldrich, St. Louis, MO, USA) supplemented with 10% FBS (MP Biomedicals, Santa Ana, CA, USA) in the presence of either recombinant 20 ng/ml human granulocyte-macrophage colony-stimulating factor (GM-CSF) (215-GM; R&D Systems, Minneapolis, MN, USA) or 50 ng/ml macrophage colony-stimulating factor (M-CSF) (216-MC; R&D Systems) for 9 days to induce differentiation to M1 or M2 Mφ, respectively [25].

### Flow cytometry

Antibodies for CD68 (FITC 333806), CD163 (PE-Cy7 333614), and CCR1 (PE 362904) were purchased from BioLegend (San Diego, CA, USA). Incubation of polarized Mφ with antibodies was performed according to the manufacturer’s protocol. M1 and M2 Mφ were defined as CD68^+^CD163^−^ and CD68^+^CD163^+^ cells, respectively. All the gates were determined using fluorescence minus one control. Data were collected on a BD FACSCanto II instrument (BD Biosciences, San Jose, CA, USA) and analyzed using FlowJo software version 10 (FlowJo, Ashland, OR, USA).

### IHC (Immunochemical) staining

Cultured cells were fixed on Nunc Lab-Tek chamber slides (Thermo Fisher Scientific, Waltham, MA, USA) with 4% paraformaldehyde. The slides and paraffinized skin specimens obtained from four patients with BD and four patients with systemic sclerosis (SSc) were deparaffinized in xylene, hydrated in ethanol, and pretreated with 10 mmol/L citrate buffer (pH 6.0). The cells and tissues were used for IHC analysis. Mouse monoclonal anti-CD68 antibody (PGM1; Dako, Carpinteria, CA, USA), anti-CD163 antibody (Leica Biosystems, Berlin, Germany), and rabbit polyclonal immunoglobulin G (IgG) anti-CCR1 antibody (GenWay Biotech, San Diego, CA, USA) at 1:100 dilution were used as primary antibodies. The REAL EnVision Detection System (Dako) was used with secondary antibodies for visualization by light microscopy [26]. To count M1 and M2 Mφ in skin lesions, CD68^+^ and CD163^+^ cells were counted in three high-power fields (magnification × 400), and the average CD68/CD163 ratio was obtained in each patient. For double-immunofluorescence staining, Alexa Fluor 555 anti-mouse IgG (Life Technologies, Carlsbad, CA, USA) and Alexa Fluor 488 anti-rabbit IgG (Life Technologies) at 1:500 dilution were used as secondary antibodies. The slides were mounted with 4′,6-diamidino-2-phenylindole (Vector Laboratories, Peterborough, UK) and imaged by fluorescence microscopy.

### Semiquantitative mRNA analysis

mRNA was extracted using the RNeasy kit (Qiagen, Hilden, Germany), and complementary DNA (cDNA) was synthesized using the SuperScript III reverse transcriptase (Life Technologies). Primers for real-time PCR of *CD163* (Hs00174705_m1), *CCR1* (Hs00174298_m1), *CCR2* (Hs00704702_s1), *IL6* (Hs00174131_m1), and *IL10* (Hs00961622_m1) were purchased from Applied Biosystems (Foster City, CA, USA). *GAPDH* (4310884E) was used as an internal control when performing multiplex PCR. The comparative cycle threshold (2^−ΔΔCt^) method was used for the analysis of duplicate or triplicate reactions [4].

### Measurements of cytokines

Lipopolysaccharide (LPS) from *Escherichia coli* (serotype 0111:B4; InvivoGen, San Diego, CA, USA) was used at 1 μg/ml. After incubation for 24 hours, the supernatants were centrifuged and stocked at − 80 °C until use. The concentrations of TNF-α, IL-6, and IL-10 were quantified using the BD Bead Assay (BD Biosciences) in accordance with the manufacturer’s protocol.

### Migration assay

Transwell migration assay was performed using 5-μm pore polycarbonate membrane (Corning Costar, Kennebunk, ME, USA). M1 and M2 Mφ were cultured on RepCell (CellSeed, Tokyo, Japan) [27]. After detachment of cells incubated at 20 °C for 30 minutes, the cells were used for the following experiments. M1 or M2 Mφ (5 × 10^4^) were applied in the upper chamber. In the lower chamber, 1% bovine serum albumin (Wako Pure Chemical Co., Osaka, Japan)-supplemented RPMI 1640 medium with or without recombinant human MIP-1α (R&D Systems) was applied. After incubation for 2 hours, cells in the upper chamber were removed with Q-Tips, and cells on the bottom side of the membrane were stained with Diff-Quik (Sysmex, Kobe, Japan). Cells were randomly counted in 25 high-power fields. The migration index was defined as the ratio between the migrated cell counts with or without MIP-1α.

### Plasticity assay

After a 9-day incubation of monocytes from BD or SSc with GM-CSF or M-CSF, polarized M1 or M2 Mφ were further incubated in the presence of GM-CSF or M-CSF for another 9 days (Fig. 4a). cDNA was obtained after the 18-day incubation.

### SNP genotyping

Genomic DNA was isolated with the DNeasy Blood & Tissue Kit (Qiagen) or BuccalQuick (Trimgen, Sparks, MD, USA). A TaqMan SNP genotyping assay was used to identify SNPs associated with expression of *IL10* (C___8828803_1_; Applied Biosystems) and *CCR1* (C___1198110_10; Applied Biosystems) [4].

### Statistical analysis

Student’s unpaired *t* test was used to test statistical significance. A *P* value of < 0.05 was considered significant. One-way analysis of variance was performed to compare three groups, except for the groups that did not contain sufficient samples suitable for analysis owing to allelic scarcity. In the latter instance, we combined heterozygous with homozygous minor alleles and analyzed them using an unpaired Student’s *t* test. Statistical analysis was performed using Prism software (GraphPad Software, La Jolla, CA, USA).

## Results

### Mφ polarization in human circulating monocytes

First, we tested the Mφ polarization protocol in peripheral monocytes purified from healthy control subjects (HC) according to a description in a previous report [25]. M1 and M2 Mφ differentiation was induced from peripheral monocytes by GM-CSF and M-CSF, respectively. The cultured cells showed distinct morphological features between the two culture systems (Additional file 1: Figure S1A and B). We confirmed that both mRNA and CD163 protein, one of the conventional M2 markers, were elevated in M2- rather than M1-cultured cells (Additional file 1: Figure S1C and D) [28]. Cytokine profiles after 24-h LPS stimulation revealed that M1-cultured cells produced a higher amount of IL-6, whereas M2- but not M1-cultured cells secreted IL-10 (Additional file 1: Figure S1E and F). Both types of cells did not synthesize a detectable level of either cytokine in the absence of LPS. These data indicate that both phenotypic features and cytokine profiles of cells derived from M1 and M2 conditions were compatible with those of M1 and M2 Mφ. Thus, we applied this protocol in the following experiments.

### CCR1 and IL-10 expression by M1 and M2 Mφ differentiated in vitro

**Fig. 1:**
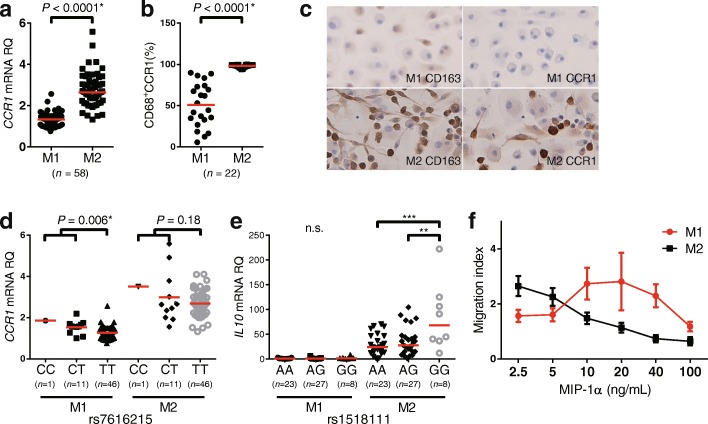
Comparison of M2 marker expression and function between M1 and M2 macrophages (Mφ) derived from healthy control subjects (HC). C-C chemokine receptor 1 (CCR1) messenger RNA (mRNA) and surface protein expression of M1 and M2 Mφ were detected using (**a**) real-time PCR and (**b**) flow cytometry, respectively. **c** IHC staining showing expression of CD163 and CCR1 in M1 and M2 Mφ (original magnification × 400). **d** *CCR1* single-nucleotide polymorphism (SNP) rs7616215 genotype and expression of *CCR1* mRNA and (**e**) *IL10* SNP rs1518111 genotype and *IL10* mRNA expression in M1 and M2 Mφ. **f** Chemotaxis of M1 (*red*) and M2 Mφ (*black*) toward macrophage inflammatory protein (MIP)-1α were determined by Transwell migration assay. The results are shown as the migration index. *Red horizontal bars* indicate median values. *P* values were determined by Student *t* test and one-way analysis of variance (**P < 0.01, ***P < 0.001). *RQ* Relative quantity

Having established the Mφ polarization protocol, we compared CCR1 expression and IL-10 production, both of which are identified as BD susceptible genes, by M1 and M2 Mφ. We found that M2 Mφ showed higher amounts of CCR1 mRNA and protein than M1 Mφ (Fig. 1a and b). Consistently, immunocytochemical analysis confirmed that CD163 and CCR1 were more abundant in M2 Mφ than in M1 Mφ (Fig. 1c).

We next looked at the association between *IL10* and *CCR1* mRNA expression and their corresponding BD-associated SNP genotypes. We found that BD risk allele was associated with decreased expression of both *CCR1* and *IL10* mRNA production in polarized Mφ. A significant expression quantitative trait loci (eQTL) effect was found only in M1 Mφ for CCR1 and IL10 for M2 Mφ (Fig. 1d and e). A recent study demonstrated that rs7616215 has a robust eQTL effect on CCR2, but not CCR1, in monocytes [29]. However, the result was not replicated in our polarized Mφ assay (Additional file 1: Figure S2A).

To examine whether the upregulated CCR1 expression is associated with enhanced chemotaxis against CCR1 ligands, we used a Transwell migration assay with gradient density of MIP-1α, a ligand for CCR1. We found that the migration of M1 Mφ into the lower chamber occurred in relatively higher MIP-1α concentrations than M2 Mφ (Fig. 1f). However, the migration of M2 Mφ into the lower chamber was more efficient at low concentrations of MIP-1α than M1 Mφ, whereas the chemotaxis was rather inhibited with higher concentrations of the chemokine (Additional file 1: Figure S3). Similar findings were reported previously in an unrelated study using this assay system [30]. These findings indicate that increased CCR1 expression contributes to sensitive chemotaxis in response to low levels of MIP-1α in M2 Mφ. These results support the notion that BD-associated *CCR1* and *IL10* loci have the capacity to alter migration and anti-inflammatory cytokine production in polarized Mφ.

### M1 and M2 Mφ in patients with BD

**Fig. 2:**
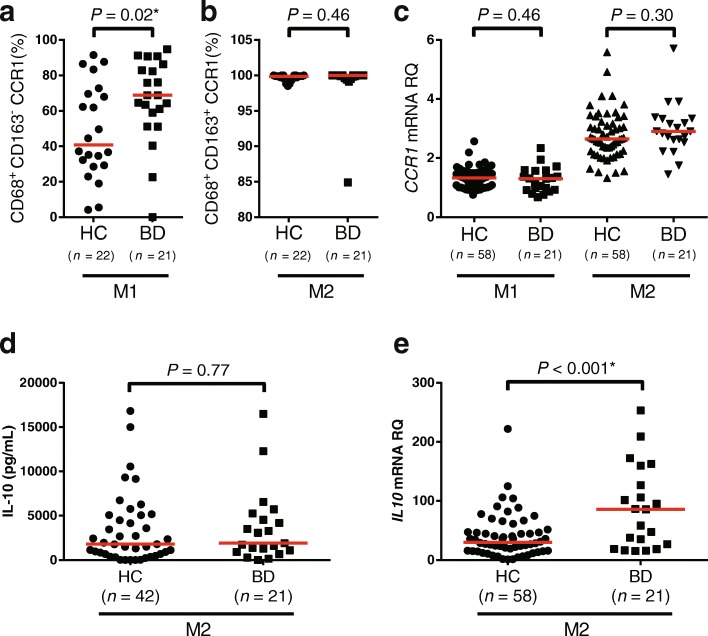
Comparison of C-C chemokine receptor 1 (CCR1) and interleukin (IL)-10 expression in macrophages (Mφ) derived from between healthy control subjects (HC) and patients with Behçet’s disease (BD). Flow cytometric analysis of CD68^+^CD163^−^CCR1^+^ (M1 Mφ) cells (**a**) and CD68^+^CD163^+^CCR1^+^ (M2 Mφ) cells (**b**) from HC and patients with BD. **c** *CCR1* messenger RNA (mRNA) expression in M1 and M2 Mφ from HC and patients with BD. **d** Supernatant IL-10 concentration of lipopolysaccharide-stimulated Mφ from HC and patients with BD. **e** *IL10* mRNA expression in M1 and M2 Mφ from HC and patients with BD. *Red horizontal bars* indicate median values. *P* values were determined by Student’s *t* test. *RQ* Relative quantity

To ask whether the CCR1 expression is affected by the disease itself, we compared the frequency of CCR1-positive M1 and M2 Mφ from HC and BD. To carry out this experiment, 21 patients with BD who fulfilled ISG criteria were enrolled. The background characteristics of the patients and HC are shown in Additional file 1: Table S1 and Table S2, respectively. The results showed that CCR1 surface expression of M1 Mφ was significantly increased in BD compared with HC (Fig. 2a), whereas no difference was detected in M2 Mφ (Fig. 2b). No significant difference between BD and HC was observed in CCR1 mRNA expression. The eQTL effects of rs7616215 on CCR1/2 mRNA and protein are shown in Additional file 1: Figure S2. The statistically significant allele-specific expression was seen only in HC.

We confirmed that no statistically significant difference in CCR1 positivity was found between treatment with or without biologics, colchicine, or prednisolone (Additional file 1: Figure S4). We also investigated the number of differentiated Mφ between HC and BD, although no significant difference was observed between the groups (Additional file 1: Figure S5).

IL-10 protein and mRNA expression in M2 Mφ from HC and BD is shown in Fig. 2d and e. *IL10* mRNA expression was elevated in BD, whereas IL-10 protein production was comparable between BD and HC. The eQTL effect of rs1518111 on IL-10 protein and mRNA is shown in Additional file 1: Figure S6. Statistically significant allele-specific expression was seen only in HC *IL10* mRNA.

### M1 and M2 Mφ in skin lesions of BD and systemic sclerosis

GWASs and our in vitro data suggested that GWAS-identified *CCR1* SNPs confer significant eQTL effects in polarized M1 Mφ, which may lead to skewed Mφ polarization balance in BD local inflammation. To prove this concept, we performed IHC staining of erythema nodosum, which was pathologically diagnosed as septal panniculitis, from the lower limbs of a treatment-naïve patient with BD. We used skin lesions of SSc as a control, which was recently reported to be M2-dominant [31].

**Fig. 3:**
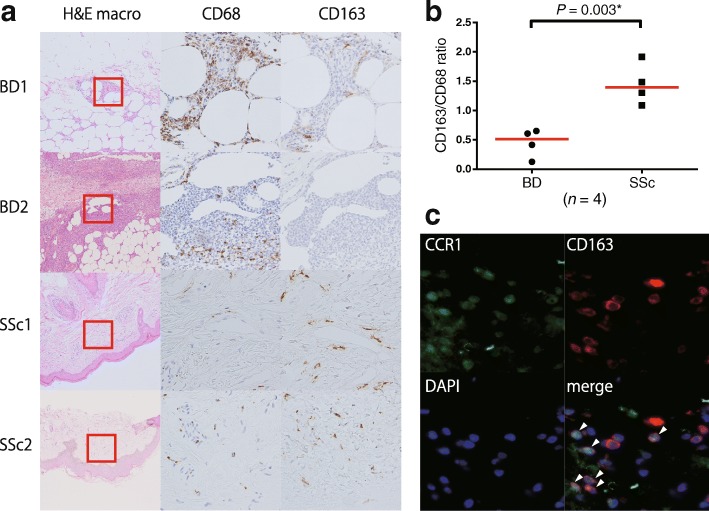
Comparison of M1 and M2 macrophages (Mφ) in Behçet’s disease (BD) and systemic sclerosis (SSc) Mφ, and immunofluorescence assay of dermal tissue of a patient with BD. **a** IHC staining of erythema nodosum from patients with BD and sclerotic dermis of patients with SSc. Antibodies against CD163 and CCR1 (original magnification × 100 [H&E staining] and × 400 [IHC staining]) were used. **b** The ratio of CD163^+^ cells (defined as M2 Mφ) and CD68^+^ cells (defined as whole Mφ) were independently counted in three high-power fields, and the mean value for each patient was calculated. **c** Immunofluorescence analysis of CCR1 (*green*) and CD163 (*red*) in BD erythema nodosum tissue. Nuclei are stained with 4′,6-diamidino-2-phenylindole (DAPI; *blue*) (original magnification × 400). *Arrowhead* indicates CCR1 and CD163 double-positive cells (merged image)

As shown in Fig. 3a, CD68^+^ cells are predominant over CD163^+^ cells in skin lesions of patients with BD compared with patients with SSc (Fig. 3b). CCR1 and CD163 double-positive cells were found in the skin lesions of patients with BD (Fig. 3c). These results suggest M1 Mφ-dominant inflammation in BD.

### Plasticity in human M1 and M2 Mφ

As shown in Additional file 1: Figure S1F, M2 Mφ have the capacity to produce IL-10, which has potent anti-inflammatory effects. Lower IL-10 production is a risk for not only BD but also ulcerative colitis [32] and other inflammatory diseases. *IL10R*-deficient patients experience oral ulceration and IBD, phenotypes resembling BD [33]. It would be of great benefit for patients with BD and other inflammatory diseases if IL-10 supplementation restored M2 Mφ function at inflammatory sites, leading to a favorable clinical outcome.

**Fig. 4:**
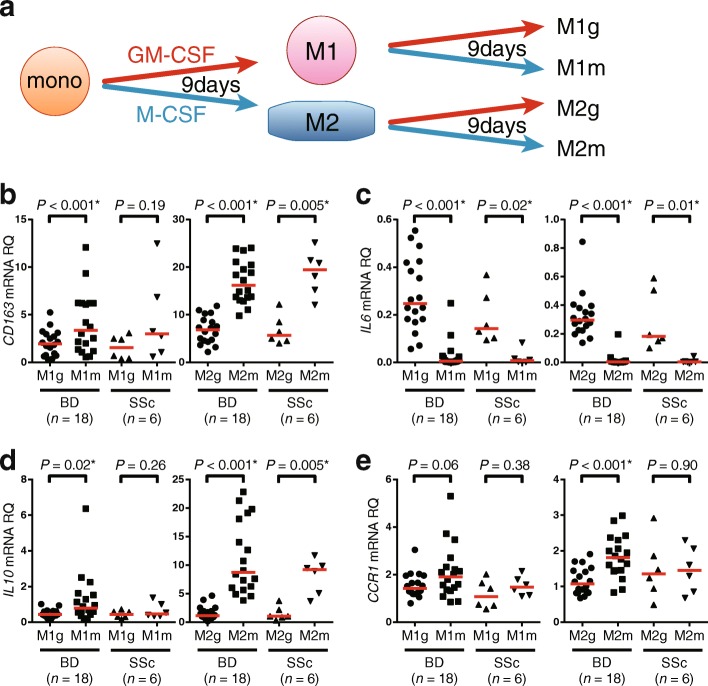
Plasticity of M1 and M2 macrophages (Mφ). **a** Protocol of plasticity assay in Behçet’s disease (BD) and systemic sclerosis (SSc) Mφ. **b–e** *CD163* (**b**), *IL-6* (**c**), *IL-10* (**d**), and *CCR1* (**e**) mRNA expression from M1g, M1m, M2g, and M2m cells are shown. *Red horizontal bars* indicate median values. *GM-CSF* Granulocyte-macrophage colony-stimulating factor, *M-CSF* Macrophage colony-stimulating factor, *RQ* Relative quantity

We tested whether established M1 Mφ could be converted into M2 Mφ in humans. To this end, the M1 and M2 Mφ that underwent 9-day in vitro polarization by GM-CSF or M-CSF were cultured for an additional 9 days in the presence of the same or other cytokines (Fig. 4a). Expression of *CD163*, *IL6*, *IL10*, and *CCR1* mRNA was measured at the end of culture (Fig. 4b–e). We found that treatment with M-CSF, but not with GM-CSF, restored expression of *IL10* mRNA with downregulation of *IL6* mRNA. On the contrary, *IL10* mRNA expression was decreased by GM-CSF in M2 Mφ. Thus, the final expression patterns of *CD163*, *IL6*, *IL10*, and *CCR1* mRNA depended on the second cytokine rather than on the first one. The results suggest that Mφ phenotypes are partly but not completely interchangeable between M1 and M2 Mφ, depending on circumstantial factors, including cytokines.

## Discussion

In the previous GWASs and subsequent genetic studies, associations of *IL10* [4, 6, 14] and *CCR1* [5, 34] loci with BD were independently replicated in multiple populations. Therefore, they are now considered bona fide loci involved in the pathogenesis of BD [35], though the pathological role remains uncertain regarding the functional basis.

The present study shows that both *IL10* and *CCR1* genes were preferentially expressed on M2 Mφ, suggesting that the disease-susceptible SNPs of the genes are responsible for impaired migration of M2 Mφ into the inflammatory lesions and reduced local synthesis of IL-10 in BD. Thus, although *IL10* and *CCR1* SNPs are independentl*y* identified as susceptible loci of BD, it is likely that their pathogenic roles are summarized into the impaired M2 Mφ function in BD.

Anti-inflammatory effects of IL-10 are mediated by HO-1, which was previously shown to be expressed on M2 Mφ preferentially [36, 37]. We have previously shown defective HO-1 synthesis to be associated with excessive expression of Toll-like receptor 4, another disease-susceptible gene product, leading to amplification of inflammation in patients with BD [23, 38]. These findings provided additional circumstantial evidence of impaired M2 Mφ functions in BD.

Although biologics targeting IL-10 would be useful for the treatment of inflammatory diseases, there are no commercially available drugs that directly manipulate IL-10 levels in humans. In animals, enhancing M2 Mφ has been shown to be protective in several SLE models [39, 40]. It is plausible that a treatment strategy could be directed at enhancement of IL-10 production or local accumulation of M2 Mφ into inflammatory lesions, contributing to the improvement of clinical outcomes of BD. TNF inhibitors may lead to a relative enrichment of M2 Mφ by inhibiting M1 Mφ function, because we have previously shown that *HO-1* mRNA expression in circulating monocytes is increased during treatment with infliximab in patients with rheumatoid arthritis [41]. Alternatively, apremilast, a phosphodiesterase 4-selective inhibitor for which an international clinical trial for BD is ongoing, stimulates the production of IL-10 and downregulates that of proinflammatory cytokines [42]. These findings suggest that clinical efficacy of these treatments is associated with corrected M1/M2 balance for BD, though direct evidence has not been provided to date.

A significant difference between BD and HC was observed in CCR1 protein expression but not in CCR1 mRNA expression. We could not detect genotype-dependent IL-10 expression differences in BD. Rather, *IL10* mRNA expression was higher in patients with BD (Fig. 2e). We posit the following possibilities:

1. Because the disease-protective GG genotype of *IL10* SNP rs1518111 and the CC genotype of *CCR1* SNP rs7616215 in patients with BD were small, the power to identify mRNA expression differences was limited in BD samples.
2. Effects of disease status or treatments could have affected IL-10 expression.
3. The sample size was too small to identify small expression differences caused by the SNP.

However, because SNPs tag common DNA haplotypes in human populations, data identified in healthy individuals would be applicable in BD pathogenesis. Further IL-10 and CCR1 transcriptional analysis is needed to clarify whether IL-10 and CCR1 expression may be dysregulated in BD.

There has been a discrepancy regarding the eQTL effect of rs7616215 genotype on CCR1/2 expression between this report and a former one [29]. We speculate the following possibilities:

1. The examined cells are different; we evaluated M1 and M2 macrophages, whereas Ishigaki et al. evaluated human peripheral monocytes.
2. The assay used to detect mRNA expression was different; we used real-time PCR, whereas Ishigaki et al. used RNA sequencing.
3. The sample size is too small to identify CCR2 expression differences in our study.

However, in our previous paper [5], we found a similar CCR1 eQTL pattern in human peripheral monocytes in samples from white Americans as well as in publicly available mRNA array data [43]. These data support our hypothesis that rs7616215 affects CCR1 mRNA expression.

We could not evaluate *CCR1* genotype dependent migration differences in the present study for the following reasons: (1) 50-ml blood samples were needed to obtain a large-enough M1 or M2 macrophage count for the Transwell migration assay, and (2) owing to large experimental variation of the Transwell migration assay of polarized macrophages, a large sample size would be necessary to detect subtle effects caused by the common SNP. However, as we have previously shown significant migration differences by rs7616215 genotypes in human monocytes [5], similar migration differences may be found in human macrophages as well.

**Fig. 5:**
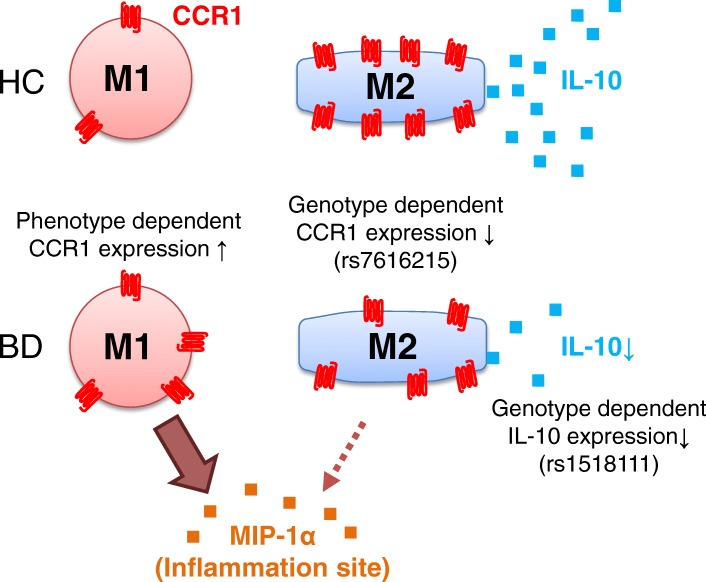
The hypothesis of C-C chemokine receptor 1 (CCR1) and interleukin (IL)-10 function in Behçet’s disease (BD) macrophages. *MIP-1α* Macrophage inflammatory protein, *HC* Healthy control subjects

Figure 5 shows our hypothesis based on the present study. M2 Mφ expressing high levels of CCR1 have the advantage of migration to inflammatory lesions, where CCR1 ligands such as MIP-1α are secreted, compared with M1 Mφ. Subsequently, the local inflammation is decreased by M2 Mφ in healthy individuals. However, the inflammatory condition would be persistent in patients with BD because M2 Mφ function is impaired by the genetic predisposition. Local accumulation of M2 Mφ is decreased owing to the reduced CCR1-dependent chemotaxis. Local IL-10 production would also be much less in patients with BD than in HC owing to the reduced number of M2 Mφ in addition to low IL-10-synthesizing capacity. Furthermore, the frequency of CCR1-positive M1 Mφ is rather increased in patients with BD compared with HC, suggesting a more efficient accumulation of M1 Mφ into MIP-1α-enriched inflammatory lesions in BD than in HC. These unexpected findings may be caused by epigenetic or environmental factors [44]. Taken together, both quantitative and functional balance between M1 and M2 Mφ shift toward M1-predominant type in inflammatory lesions, resulting in a vicious cycle of local inflammation. We added our new hypothesis to the previously proposed schema representing the pathogenesis of BD (Additional file 1: Figure S7) [45, 46].

Phenotypic plasticity of matured Mφ is an important issue for future study. Our data indicated that Mφ function was modified by M-CSF or GM-CSF at least in vitro. Particularly, we confirmed that in vitro polarized M1 Mφ restored IL-10 synthesis capacity in the presence of M-CSF. Even such partial modification of Mφ function would be beneficial for patients with BD.

This study has some limitations. First, most of the data were obtained from in vitro experiments, which might not fully reflect the in vivo biological events in patients with BD. Second, because biopsies of affected lesions in BD are not routinely done for the diagnosis of BD, only small numbers of skin biopsy specimens could be evaluated. In addition, the timing of skin biopsy in the disease course may influence the balance between M1 and M2 Mφ. Finally, most of the patients with BD were receiving some of the therapeutic agents, which might have affected the results of in vitro culturing. However, the 9-day in vitro polarization protocol may have minimized the influence of the therapeutic agents, as shown in Additional file 1: Figure S4. Nonetheless, our data reveal the difference in CCR1 expression of M1 Mφ between patients with BD and HC.

## Conclusions

The present study shows that BD-associated *CCR1* and *IL10* loci are responsible for defective M2 Mφ, resulting in skewed M1 Mφ polarization in BD. Our data suggest that phenotypic plasticity is partially conserved in matured M1 and M2 Mφ. Strategies to correct the imbalance between M1 and M2 Mφ or to modify Mφ function would be promising for patients with BD in the future.

## Additional file

Additional file 1: **Figure** **S1.** Comparison of phenotypic features and cytokine profiles between M1 and M2 cultured cells. **Figure S2.** eQTL effect of rs7616215 on CCR2 and CCR1. **Figure S3.** Detailed results of chemotaxis of M2 Mφ toward various concentrations of MIP-1α. **Figure S4.** Differences in CCR1 positivity (fluorescence-activated cell sorting) between treatments. **a** Biologics. **b** Colchicine. **c** Prednisolone. **Figure S5.** The number of differentiated Mφ in vitro in HC and BD. **Figure S6.** eQTL effect of rs1518111 on IL-10 protein and mRNA. **Figure S7.** Schema showing our proposed immunological responses in BD. **Table S1.** Characteristics of patients with BD who participated in the study. **Table S2.** Characteristics of HC study participants. (DOCX 9663 kb)

## Abbreviations

BD: Behçet’s disease
CCR: C-C chemokine receptor
CD: Cluster of differentiation
cDNA: Complementary DNA
DAPI: 4′,6-Diamidino-2-phenylindole
eQTL: Expression quantitative trait loci
GAPDH: Glyceraldehyde 3-phosphate dehydrogenase
GM-CSF: Granulocyte-macrophage colony-stimulating factor
GWAS: Genome-wide association study
HC: Healthy control subjects
HO: Heme oxygenase
IBD: Inflammatory bowel disease
IgG: Immunoglobulin G
IL: Interleukin
ISG: International Study Group
LPS: Lipopolysaccharide
M-CSF: Macrophage colony-stimulating factor
MIP: Macrophage inflammatory protein
mRNA: Messenger RNA
Mφ: Macrophages
PBMC: Peripheral blood mononuclear cells
SLE: Systemic lupus erythematosus
SNP: Single-nucleotide polymorphism
SSc: Systemic sclerosis
Th: Helper T cell
TNF: Tumor necrosis factor
