# Supplementary material for: GWAS-identified CCR1 and IL10 loci contribute to M1 macrophage-predominant inflammation in Behçet’s disease
Source: Arthritis Res Ther. 2018 Jun 12;20:124. doi: 10.1186/s13075-018-1613-0 (PMC5998575; doi:10.1186/s13075-018-1613-0)
Supplement: Supplementary file 1 — Figure S1. Comparison of phenotypic features and cytokine profiles between M1 and M2 cultured cells. Figure S2. eQTL effect of rs7616215 on CCR2 and CCR1. Figure S3. Detailed results of chemotaxis of M2 Mφ toward various concentrations of MIP-1α. Figure S4. Differences in CCR1 positivity (fluorescence-activated cell sorting) between treatments. a Biologics. b Colchicine. c Prednisolone. Figure S5. The number of differentiated Mφ in vitro in HC and BD. Figure S6. eQTL effect of rs1518111 on IL-10 protein and mRNA. Figure S7. Schema showing our proposed immunological responses in BD. Table S1. Characteristics of patients with BD who participated in the study. Table S2. Characteristics of HC study participants. (DOCX 9663 kb) [file 13075_2018_1613_MOESM1_ESM.docx]

Additional file 1

**Figure S1** Comparison of phenotypic features and cytokine profile between M1 and M2 cultured cells.

M1 and M2Mφ differentiation induced from purified peripheral monocytes were cultured for 9 days in the presence of M-CSF and GM-CSF, respectively. Light scope morphology of (A) M1 and (B) M2 cultured cells were shown (x400, Diff-Quik). CD163 mRNA and surface expression were determined by (C) real-time PCR and (D) flow cytometry, respectively. Amounts of (E) IL-6 and (F) IL-10 in the supernatants from M1 and M2 cultured cells with or without LPS (ng/ml) stimulation for 24 hours are determined by bead assay. Red horizontal bars indicate median value.

**Figure S2** eQTL effect of rs7616215 on CCR2 and CCR1.

The eQTL effect of rs7616215 SNP genotypes on (A) *CCR2* mRNA expression in HC polarized Mφ and (B) *CCR1* mRNA expression in BD polarized Mφ were shown. The eQTL effect of rs7616215 SNP genotypes on CCR1 protein expressions were shown in C & D. Red horizontal bars indicate median value. *P* values were determined by Student *t*-test.

**Figure S3** The detail result for chemotaxis of M2 Mφ toward various concentration of MIP-1α.

**Figure S4** The difference in CCR1 positivity (FACS) between treatment (biologics (A), colchicine (B) or prednisolone (C)).

Red horizontal bars indicate median value.

**Figure S5** The number of in *vitro* differentiated Mφ in HC and BD.

Red horizontal bars indicate median value.

**Figure S6** eQTL effect of rs1518111 on IL-10 protein and mRNA.

Red horizontal bars indicate median value.

**Figure S7** Schema showing our proposing immunological responses in BD.

Tregs: regulatory T cells, APCs: antigen presenting cells, Neut: neutrophils.

**Table S1** Characteristics of BD patients participated in the study.

| *n* = 21 | (%) |
| --- | --- |
| Male | 14 (66.7) |
| Age* | 49 [43, 57] |
| Ethnic Japanese | 21 (100) |
| Disease duration (years)* | 7.5 [3, 23.3] |
| Symptoms  Aphthous stomatitis | 21 (100) |
| Skin lesions | 20 (95.2) |
| Uveitis | 18 (85.7) |
| Genital ulcers | 14 (66.7) |
| Gastrointestinal lesions | 3 (14.3) |
| Vascular lesions | 1 (4.8) |
| CNS lesions | 3 (14.3) |
| Arthritis | 9 (42.9) |
| Epididymitis | 0 (0) |
| Treatments  Prednisolone | 3 (14.3) |
| Colchicine | 14 (66.7) |
| Methotrexate | 2 (9.5) |
| Infliximab | 12 (57.1) |
| Adalimumab | 2 (9.5) |

*median [IQR]

**Table S2** Characteristics of HC study participants.

| *n* = 58 | (%) |
| --- | --- |
| Male | 45 (77.6) |
| Age* | 35 [30, 38]** |
| Ethnic Japanese | 58 (100) |

*median [IQR]

** *n* = 38. Data for 20 participants unavailable due to privacy restrictions.
